# Progranulin derived engineered protein Atsttrin suppresses TNF-α-mediated inflammation in intervertebral disc degenerative disease

**DOI:** 10.18632/oncotarget.22766

**Published:** 2017-11-29

**Authors:** Hong Ding, Jianlu Wei, Yunpeng Zhao, Yi Liu, Lian Liu, Lei Cheng

**Affiliations:** ^1^ Department of Orthopaedics, Qilu Hospital of Shandong University, Jinan, China

**Keywords:** Atsttrin, TNF-α, progranulin, intervertebral disc degeneration, inflammation

## Abstract

Atsttrin, an engineered molecule composed of three fragments of progranulin(PGRN), exerts comparable anti-inflammation ability. Intervertebral disc degeneration (IDD) is involved in inflammation in which TNF-α plays a key role. This study aims to examine the effect and the mechanism of Atsttrin in the pathogenesis of intervertebral disc degeneration. For this purpose, we took advantage of murine and human intervertebral disc (IVD) and examined the expression of TNF-α in IVD tissues using immunohistochemistry and TNF-α level in peripheral sera by ELISA assay. Moreover, murine IVD was taken to undergo the Safranin O and HE staining. Furthermore, primary human nucleus pulposus cells were used for immunohistochemistry staining, fluorescent staining, Western Blot, ELISA assay and RT-PCR assay. Herein we found TNF-α expression was elevated in intervertebral disc and peripheral sera in patients with IDD. Interestingly, Atsttrin effectively inhibited TNF-α-mediated catabolism in murine disc by *ex vivo* study. TNF-α-induced inflammatory cytokines were strongly reduced in presence of Atsttrin in primary human disc. Mechanism study indicated Atsttrin protected against intervertebral disc degeneration by inhibiting TNF-α-induced inflammation. These findings show that Atsttrin is a potential molecular target for disc degenerative diseases.

## INTRODUCTION

Intervertebral disc degeneration(IDD) is an irreversible degenerative disease which is very common in the elderly, characterized by disc inflammation, endplate degeneration, vertebral osteophyte formation, spinal canal stenosis [1, 2]. The clinical manifestation of IDD include low back pain, radiating ache in lower extremity, numbness and weak of low limb [3], strongly affect the patient's daily life. So far there has no drugs and effective treatment can reverse the pathogenesis of intervertebral disc degeneration, and surgery is the final treatment for these diseases.

Tumor necrosis factor-α (TNF-α) plays an important role in the process of intervertebral disc degeneration, and it also be the key reason for the low back pain [4, 5]. Serum level of TNF-α is relative to the pain threshold in IDD patients [6]. TNF-α stay at the peak of the inflammatory cascade response [7, 8]. Even slight change of TNF-α causes great changes of the downstream inflammatory factor such as IL-1β, MMP-13 and iNOS. Targeting TNFα pathways has been proven to be highly successful for treatment of many autoimmune diseases [2, 3].

Progranulin, which is also known as PGRN, is a growth factor which has a unique structure with “beeds-on-a-string”. PGRN is expressed in various cells and plays a critical role in various physiological and pathological processes, including wound healing, tumorigenesis and inflammation [9–11]. Atsttrin (antagonist of TNF-TNFR signaling via targeting to TNFR) is an engineered protein derived from PGRN, which is constituted of half-units of granulins A, C, and F plus linkers P3, P4, and P5 [12]. Studies indicated the engineered protein Atsttrin has similar functions with PGRN in many physiological processes. Additionally, Atsttrin has some advantages over PGRN such as longer half-life, higher efficacy, lower molecular weight and no oncogenic effects [13]. Recently, studies indicated Atsttrin exhibited protective effect in several diseases. Specifically, Atsttrin was reported to have therapeutic effect in inflammatory arthritis [12, 13]. Furthermore, Atsttrin exhibited therapeutic effect in inflammatory bowel disease [14]. Moreover, Atsttrin exerts protective effect in bone healing and degenerative osteoarthritis through inhibiting TNF-α signaling [15].

Given the indication that previous study demonstrated that loss-of-PGRN accelerated degeneration in the aging mice model and the importance of Atsttrin’s anti-inflammation ability, in this study, we determined to examine whether Atsttrin as PGRN-derived engineered molecule, has a protective effect in intervertebral disc degeneration as well as the molecular mechanism involved.

## RESULTS

### TNF-α is increased in degenerative IVD and peripheral serum

**Figure 1:**
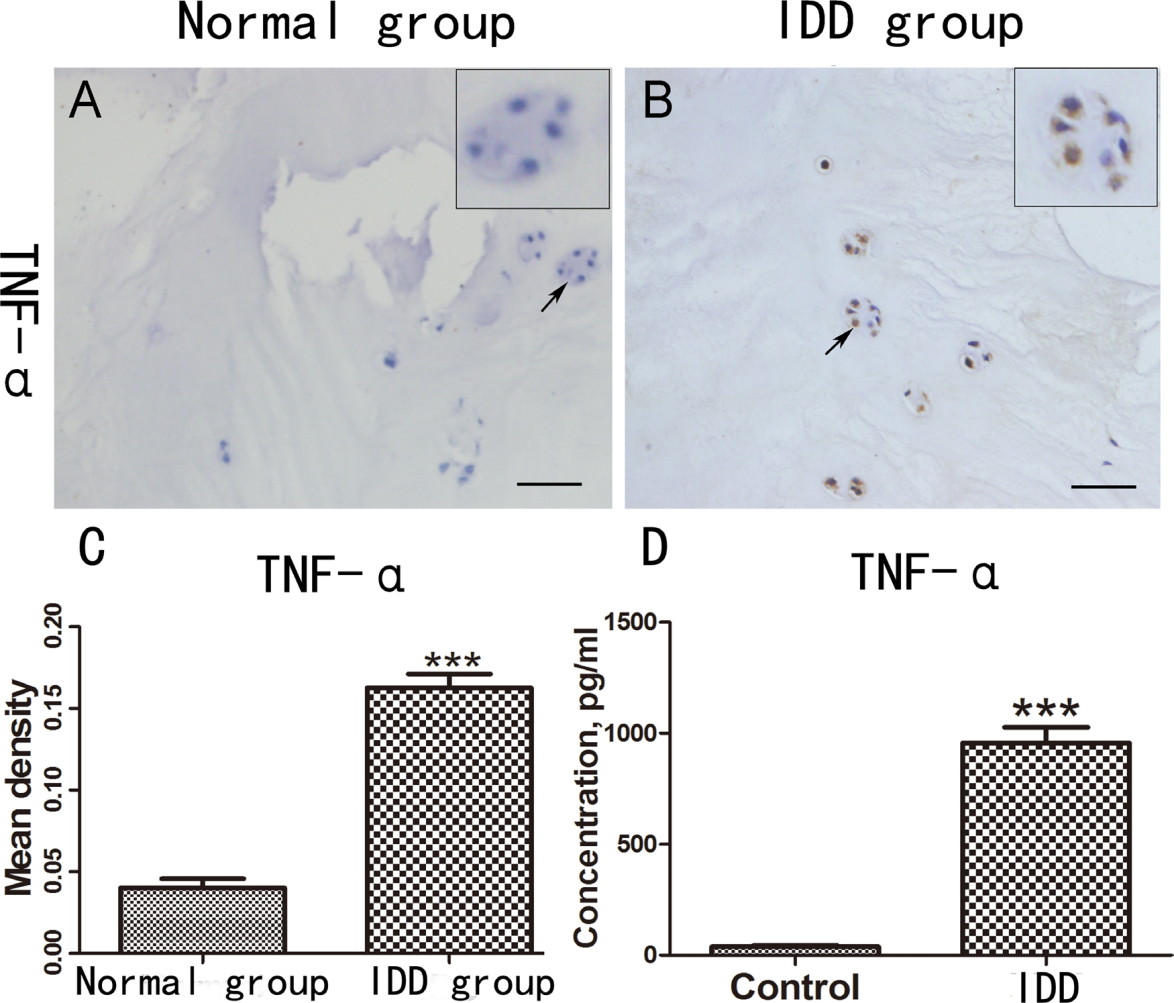
TNF-α is increased in degenerative IVD and peripheral serum **(A-B)** Expression of TNF-α in the IVD tissues of normal people and patients with IDD was detected by the immunohistochemical staining. **(C)** Mean density of the normal group and IDD group based on the result of immunohistochemical staining. **(D)** The different serum level of TNF-α in the normal people (N=10) and the patients with IDD (N=40), Scale bar=100 um. The values are mean ± SEM of at least 3 independent experiments; ^***^P<0.001.

To investigate expression of TNF-α in the pathogenesis of IDD, we performed immunohistochemistry staining in IVD tissues from normal people and patients with IDD respectively. As shown in Figure 1A & 1B, TNF-α expression in disc of IDD was remarkably increased compared to normal group (IDD group increased 310% compared with normal group, Figure 1C). Furthermore, to determine the level of TNF-α in peripheral blood of the normal people and the people with IDD, the serum were collected and ELISA assay was performed. As demonstrated in Figure 1D, TNF-α level was significantly up-regulated in IDD patients. Taken together, TNF-α is increased in the pathogenesis of IDD.

### Atsttrin exhibited therapeutic effect in murine disc *ex vivo*

**Figure 2:**
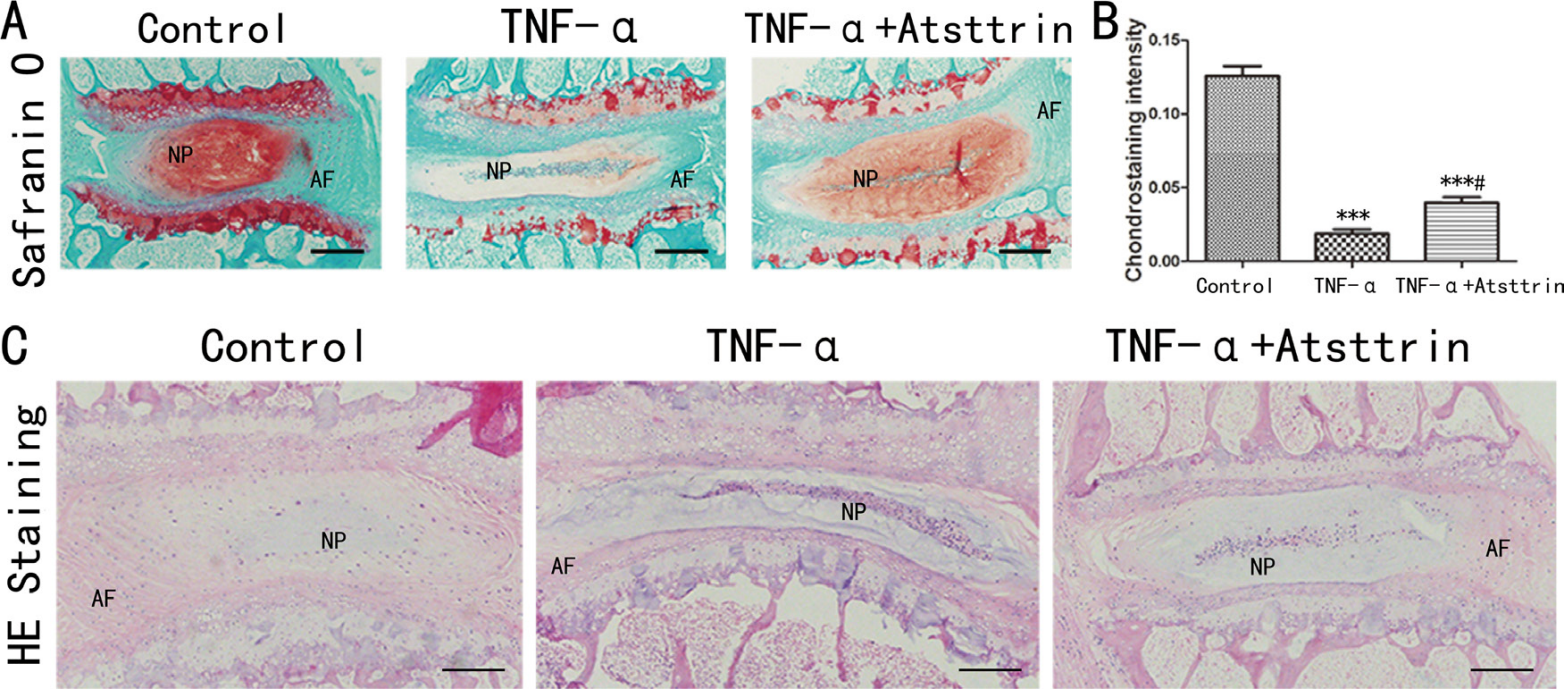
Atsttrin exhibited therapeutic effect in murine disc *ex vivo* **(A)** After culture the mouse IVD with TNF-α (50 ng/ml) in the presence or absence of Atsttrin (1 ug/ml) respectively and Safranin O staining was performed. **(B)** Chondrostaining density analysis shown Atsttrin attenuated the loss of cartilage tissue in IVD induced by TNF-α. **(C)** After culture the mouse IVD with TNF-α (50 ng/ml) in the presence or absence of Atsttrin (1 ug/ml) respectively and HE staining was performed. TNF-α accelerated the loss of the height and nucleus pulposus in IVD, while co-cultured with Atsttrin alleviated this structural degeneration. Abbreviation: NP: nucleus pulposus; AF: annulus fibrosus. Scale bar=200 um. The values are mean ± SEM of at least 3 independent experiments; ^***^P<0.001 VS control group, ^#^P<0.05 VS TNF-α treatment group.

Given the finding that TNF-α is increased in the pathogenesis of IDD and the importance of TNF-α in IDD process, to further determine whether Atsttrin could suppress TNF-α-mediated catabolism in disc, we isolated murine disc and did *ex vivo* study. For this purpose, we cultured the mice disc with or without 50 ng/ml TNF-α in the presence or absence of 1ug/ml Atsttrin for 7 days. Followed by Safranin O staining and HE staining. As is shown in Figure 2A, TNF-α strongly induced cartilage loss while in the presence of Atsttrin remarkably restored cartilage in the endplates and NP of the disc. Furthermore, statistical analysis indicated cartilage significantly decreased in the nucleus area (Figure 2B). However, Atsttrin effectively inhibited TNF-α-induced nucleus catabolic metabolism. We also performed HE staining, as is shown in Figure 2C, TNF-α accelerated the loss of the disc height and nucleus pulposus cells in IVD, while co-cultured with Atsttrin alleviated this structural degeneration. The result suggested that Atsttrin attenuated TNF-α-mediated catabolism.

### Atsttrin reduced TNF-α-induced inflammatory cytokines in human nucleus

**Figure 3:**
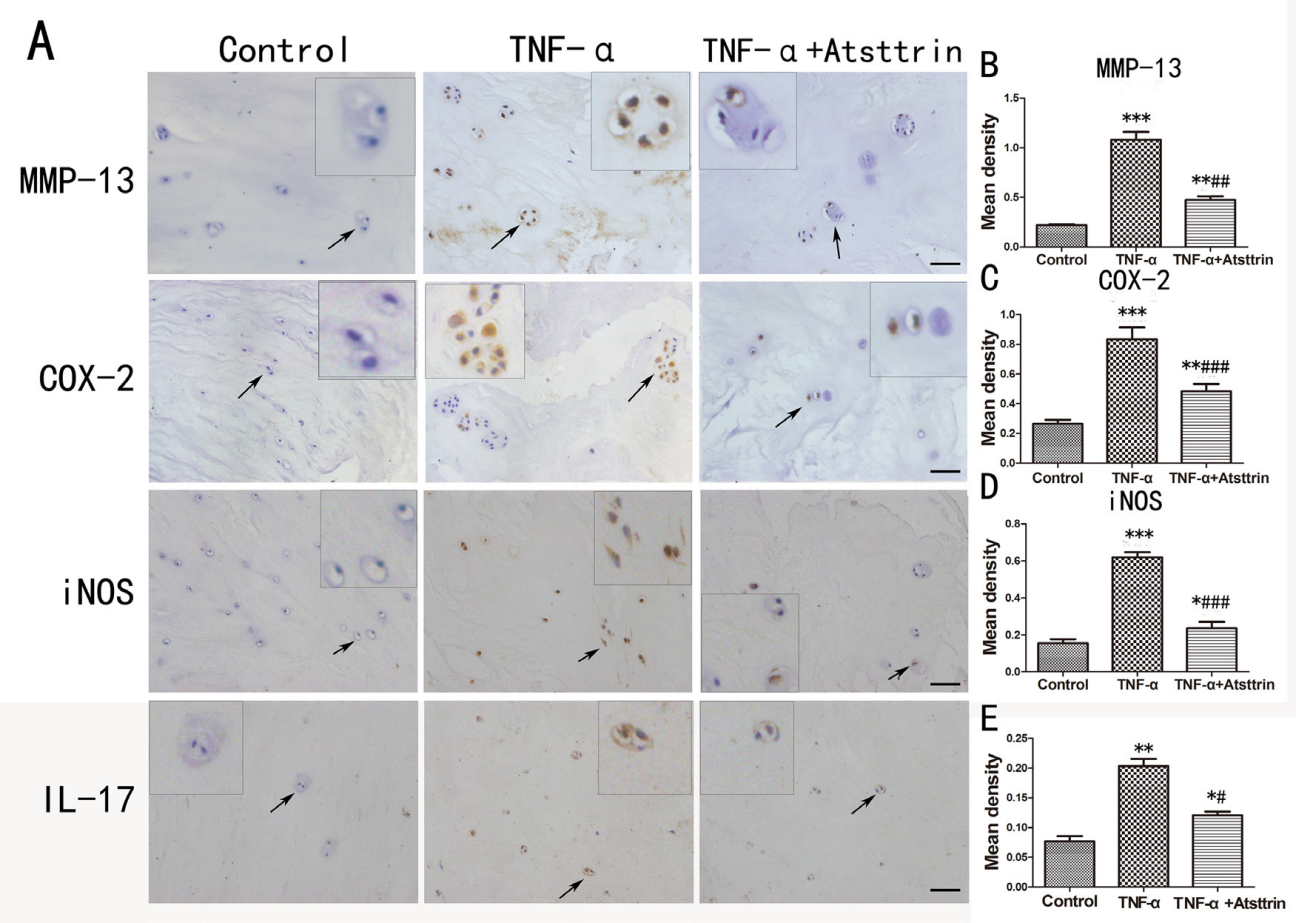
Atsttrin reduced TNF-α-induced inflammatory cytokines in human nucleus **(A)** The immunohistochemistry of MMP-13, COX-2, iNOS and IL-17 in the human IVD tissues cultured TNF-α (50 ng/ml) in the presence or absence of Atsttrin (1 ug/ml) respectively. Scale bars=100 um. **(B, C, D, E)** Quantitative analysis of the positive area of MMP-13, COX-2, iNOS and IL-17 in different groups based on the result of immunohistochemical staining. The values are mean ± SEM of at least 3 independent experiments, ^*^P<0.05, ^**^P<0.01, ^***^P<0.001 VS control group. ^#^P<0.05, ^##^P<0.01, ^###^P<0.001 VS TNF-α treatment group.

**Figure 4:**
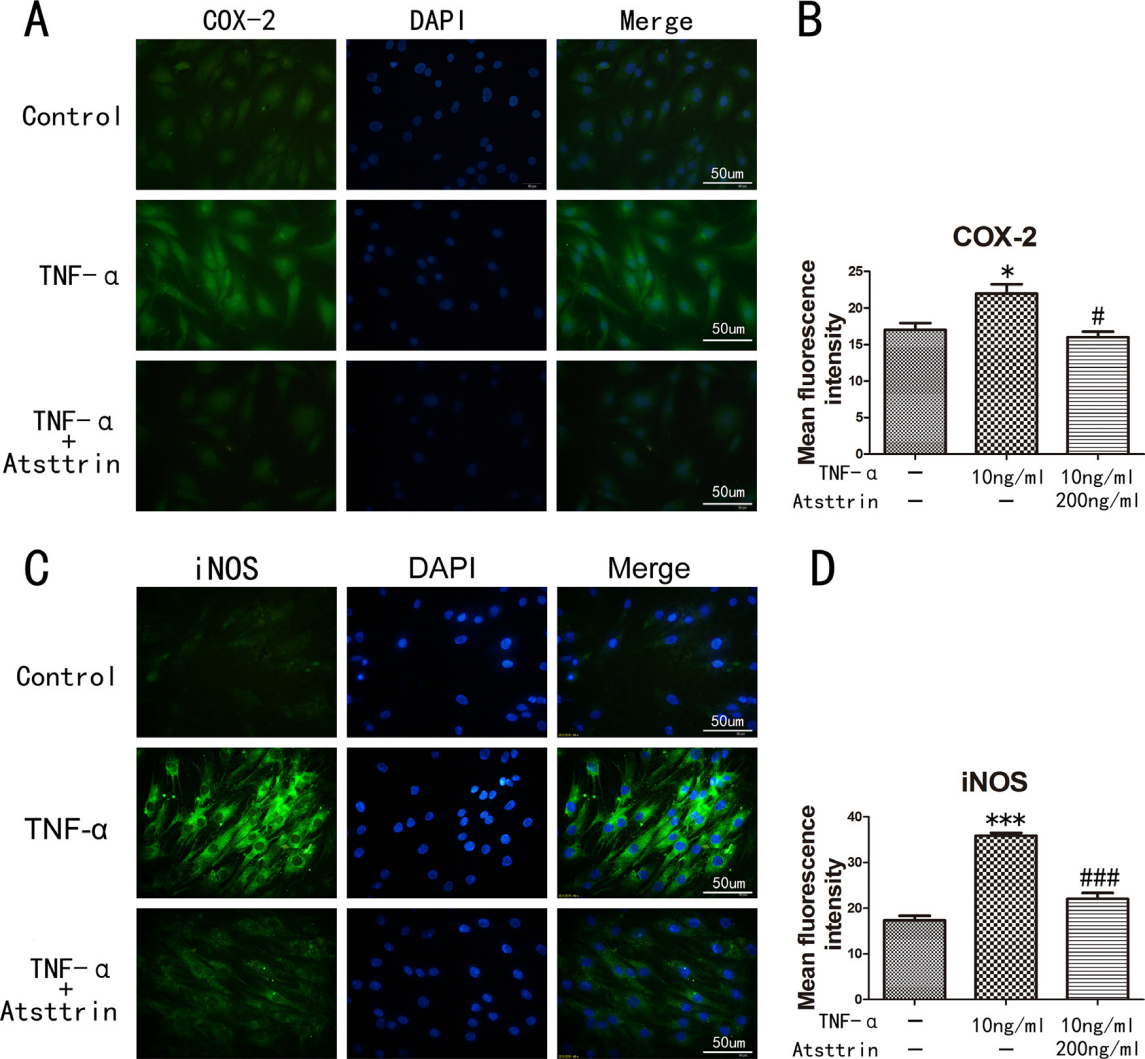
Inflammatory cytokines in human nucleus cells detected by immunofluorescent staining **(A, C)** Expression of iNOS and COX-2 in human NP cells detected by the immunofluorescence. **(B, D)** Analysis of the mean fluorescence intensity of COX-2 and iNOS according to the result of immunofluorescence results. The NP cells was treated with TNF-α (10ng/ml) in the presence or absence of Atsttrin (200 ng/ml), scal bars=50 um. ^*^P<0.05, ^***^P<0.001, VS control group. ^#^P<0.05, ^###^P<0.001, VS TNF-α treatment group.

To further determine the mechanism of Atsttrin in the process of IDD, we took advantage of primary human intervertebral disc tissues in the *ex vivo* study. It’s widely accepted that TNF-α plays a critical role in the intervertebral disc degeneration process. In addition, TNF-α-induced inflammatory cytokine such as IL-17, IL-1, COX-2 and iNOS could cause further destruction. To investigate whether Atsttrin could inhibit TNF-α-induced inflammation in human disc, we cultured the nucleus tissues with or without TNF-α in presence or absence of Atsttrin for 7 days. After harvested the tissues, immunohistochemistry staining was performed for the expression of MMP-13, COX-2, iNOS and IL-17, As is illustrated in Figure 3A, expression of MMP-13, COX-2, iNOS and IL-17 were highly elevated in presence of TNF-α which is in line with previous studies. Furthermore, as demonstrated in Figure 3B-3E, statistical analysis of the immune-staining intensity indicated that TNF-α significantly elevated expression of MMP-13, COX-2, iNOS and IL-17 up to 299%, 207%, 389% and 225%, respectively. However, as shown in Figure 3A, Atsttrin effectively reduced TNF-α-induced inflammatory cytokines in human disc. Specifically, expression of MMP-13, COX-2, iNOS and IL-17 was decreased about 62%, 42% 56% and 50%, respectively (Figure 3B-3E). To further confirm our findings, we isolated primary human nucleus cells and cultured with or without TNF-α in presence or absence of Atsttrin for 2 days. After 2 days’ incubation, we harvested and fixed the cells, followed by immunofluorescent staining. As indicated in Figure 4A & 4C, the expression of inflammatory cytokines, including COX-2 and iNOS were remarkably up-regulated after TNF-α treatment in the primary human nucleus cells. However, additional use of recombinant Atsttrin effectively reduced the expression of these molecules. Statistical analysis showed Atsttrin inhibited TNF-α-induced inflammatory cytokines *in vitro* (Figure 4B & 4D). Above all, Atsttrin alleviated TNF-α-mediated inflammation by reducing expression of TNF-α-induced downstream molecules.

### Atsttrin protects against IDD via inhibiting TNF-α signaling

**Figure 5:**
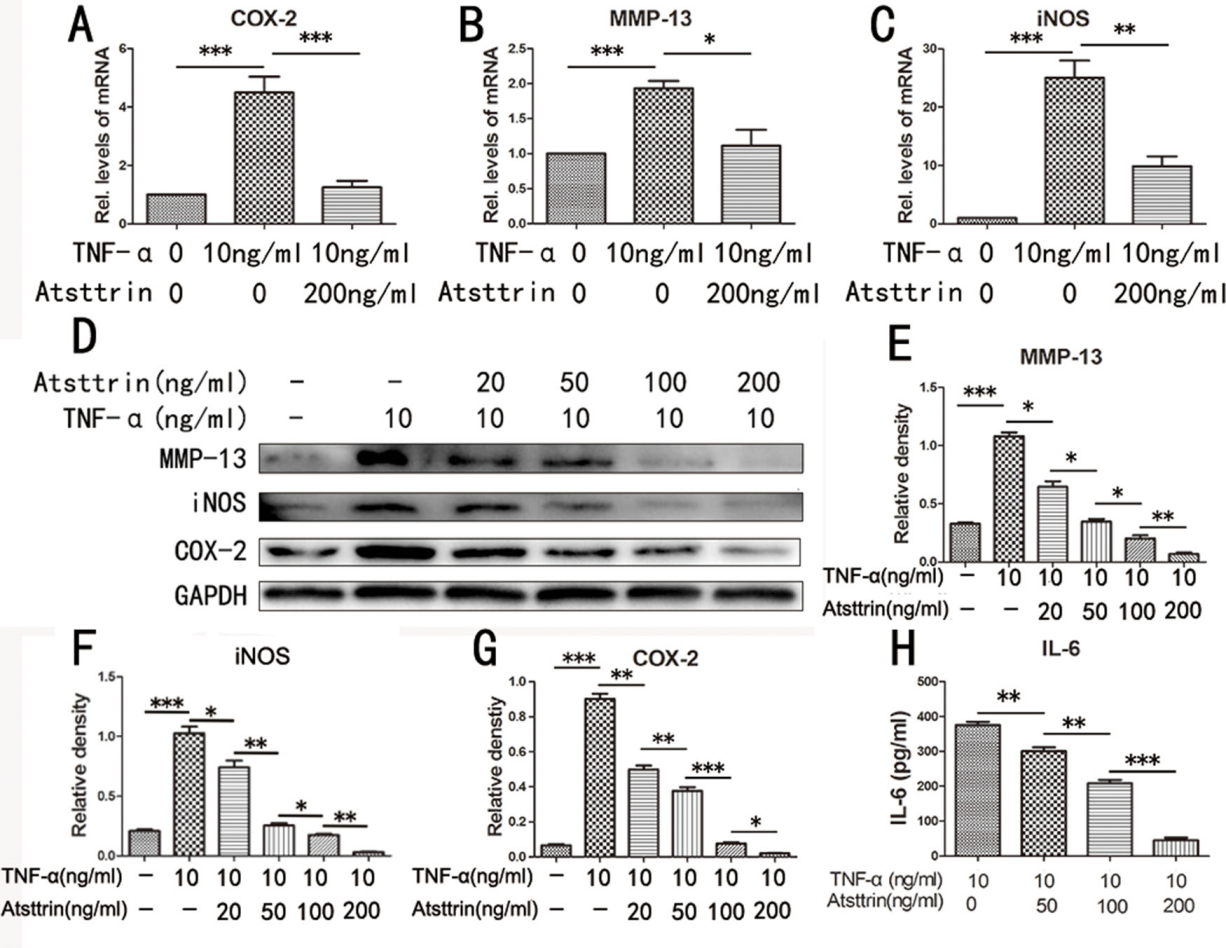
Atsttrin protects against IDD via inhibiting TNF-α signaling **(A, B, C)** MMP-13, iNOS and COX-2 mRNA expression level was detected by RT-PCR, the mRNA expression of MMP-13, iNOS and COX-2 was significant increase in the TNF-α (10 ng/ml) treatment group compared with control group, after co-culture with Atsttrin (200 ng/ml), these molecules’ transcriptional level were significantly reduce. **(D)** The protein level of MMP-13, COX-2 and iNOS in the human nucleus pulposus cells which co-culture with TNF-α in the presence or absence of different concentration of Atsttrin (20, 50, 100, 200 ng/ml). **(E, F, G)** Mean density of immunoblot of MMP-13, COX-2 and iNOS at different concentration of Atsttrin. **(H)** Secretion of IL-6 in different groups detected by ELISA. The values are mean ± SEM of at least 3 independent experiments, ^*^P<0.05, ^**^P<0.01, ^***^P<0.001.

Given the finding that Atsttrin has a therapeutic effect in IDD process and the importance of Atsttrin’s anti-TNF-α capacity, we next determined the mechanism of Atsttrin in the pathogenesis of IDD. To address this issue, we harvested the nucleus cells from human lumbar intervertebral disc and cultured the cells with 10 ng /mL TNF-α in presence or absence of 200 ng/mL Atsttrin for 6 hours. Then the total RNA was extracted and followed by Real-time PCR assay. As indicated in Figure 5A-5C, the transcriptional levels of MMP-13, COX-2 and iNOS were significantly increased in TNF-α treated group compared to the control group. While Atsttrin significantly reduced the transcriptional level of these molecules. Besides RNA transcription, we next analyzed the protein expression. In detail, we cultured the primary human nucleus cells with 10 ng /mL TNF-α in presence or absence of different concentrations of Atsttrin (0, 20, 50, 100, 200 ng/ml) for 48 hours. Supernatant and cells were collected. Total protein was extracted from cells and western blot was performed for COX-2, MMP-13 and iNOS. As revealed in the Figure 5D, Atsttrin inhibited TNF-α induction of COX-2, MMP-13 and iNOS in a dose-dependent manner (Figure 5E-5G). Moreover, as is shown in Figure 5H, the level of IL-6 in the supernatant is elevated in TNF-α treated group comparison with control group while Atsttrin could inhibited the TNF-α’s effect in a concentration-dependent manner. Collectively, Atsttrin protects against IDD through inhibiting TNF-α pathway.

## DISCUSSION

Intervertebral disc degeneration (IDD) is one of the most popular degenerative diseases which highly affect people’s daily life [2]. So far, only symptom release or modified drugs are available. However, no drugs are commercial available to cure this disease. The final outcome usually is surgery. Unfortunately, surgery accompany with high cost and uncertain complication. So clinically it’s very urgent and helpful for discovering a new alternative for treating IDD.

It’s widely accepted that inflammation plays a critical role in the pathogenesis of IDD [16, 17]. In the process, the inflammatory cytokines, such as TNF-α, IL-1, IL-17, COX-2 and iNOS are significantly increased in IDD patients [18–20]. The inflammatory molecules mediate inflammation and cause disc destruction [18, 21]. Moreover, the inflammatory molecules induce inflammatory cascade and destroy the matrix of the disc, further mass the disc. Nowadays, more and more studies focused on the anti-inflammation in IDD and achieved excited indications [22, 23].

Among these molecules, TNF-α attract researchers’ attention owing to its peak site of the inflammatory cascade [8, 24]. Previous studies indicated that TNF-α could accelerate intervertebral disc degeneration, promote adjacent bone absorb and enhance endplate degeneration [21, 25]. Furthermore, evidence showed that TNF-α is the base of inflammation in IDD process [26]. Clinical study showed that the level of TNF-α was related to low back pain in patients [4, 27, 28]. Higher TNF-α level indicated suffering more pain. Additionally, anti-TNF-α drugs exhibited a therapeutic effect in IDD process [29]. Mechanism study revealed TNF-α mess the disc through at least two ways. A) directly induce downstream inflammatory molecules, including IL-17, IL-6, to cause and enhance the local and systemic inflammation. B) mediate the expression of matrix metalloproteinase, disable the stability of the disc construction. We previously reported that TNF-α promoted CCL20 recruit TH17 cells in nucleus cells, causing increased secretion of IL-17 [30]. In addition, IL-17 mediated inflammation in nucleus cells through JNK/c-Jun signaling [31]. Collectively, given the importance of TNF-α in regulating of IDD, it’s of high innovation for discovering a new alternative medicine which targeting TNF-α pathway in IDD pathogenesis.

Progranulin, also known as PGRN, is a growth factor with potent anti-inflammation ability. PGRN consists of seven and half units (P-G-F-B-A-C-E-D) [32]. While P is a half unit and others are full unit. The unit is called granulin(GRN). Interesting, PGRN is considered as anti-inflammation factor while GRN is thought as pro-inflammation factor [33]. It’s well known that PGRN effectively interfere TNF-α signaling by completely binding to the TNF-α receptors (TNFRs) [12, 34]. On the basis of PGRN’s unique structure, Tang et al discovered an engineered protein which is named Atsttrin [12]. Atsttrin consists of half unit of F-A-C and their linkers (1/2F-1/2A-1/2C) [12, 35, 36]. Similar to PGRN, Atsttrin retains the binding affinity with TNFRs [37]. However, Atsttrin has some advantages over PGRN such as longer half-life, higher efficacy, lower molecular weight and no oncogenic effects [13]. These specific advantages imply Atsttrin may have more insights in dealing with inflammatory diseases.

Previous study indicated that Atsttrin had a therapeutic effect in the inflammatory arthritis mice model in which TNF-α plays a predominant role [12–14]. In addition, Atsttrin effectively inhibited TNF-α-induced osteoclastogenesis. Furthermore, in bone healing process, Atsttrin exerted a therapeutic effect by interfering with TNF-α signaling [38]. Recent study indicated Atsttrin was an alternative for the treatment of inflammatory bowel disease in the mice model [14]. Despite the anti-catabolic ability, it’s reported that overexpression of Atsttrin promoted cartilage repair [13]. Given the importance of TNF-α in IDD process and Atsttrin’s potent anti-inflammatory ability, we determined the role of Atsttrin in IDD.

In the present study, we found TNF-α was up-regulated in local nucleus cells and peripheral blood [28, 39]. This finding is in line with previous studies. To test the role of Atsttrin in IDD process, we performed *ex vivo* study by using murine intervertebral disc. Herein, we found additional use of TNF-α accelerated cartilaginous tissue loss in the murine intervertebral disc. Additionally, TNF-α indeed affected the construction of intervertebral disc, however, in present study we did not see the osteophyte formation which may due to the less duration of treatment or the different microenvironments between *ex vivo* and *in vivo*. Intervertebral disc is full of matrix (aggrecan and collagen II)), in the pathogenesis of IDD, inflammatory cytokines penetrated and cause destruction. Metalloproteinase 13 (MMP-13) is the main enzyme which digesting type II collagen (Col II) [40], causing matrix distortion. We found TNF-α indeed increased the expression of MMP-13 in the human nucleus tissues, while additional use of recombinant Atsttrin strongly reduced MMP-13’s expression, slowing down the matrix destruction. Furthermore, it’s well known that TNF-α directly induce iNOS and COX-2, and they are regarded as the severity maker as inflammation [41]. We confirmed this finding in the human nucleus tissues and interestingly we found Atsttrin effectively inhibited these inflammatory molecules. Besides the *ex vivo* study, we confirmed our findings by *in vitro* assay. Fluorescent staining indicated in the human nucleus cells, TNF-α remarkably increased the expression of iNOS and COX-2, however, iNOS and COX-2’s expressions were significantly decreased in presence of Atsttrin. Given the findings that recombinant Atsttrin has a potential therapeutic effect in the intervertebral disc degeneration, and the clue that Atsttrin exerted a protective effect via targeting TNF-α pathway in the inflammatory arthritis mice model, we next determined that Atsttrin inhibited this pathogenesis through interfering with TNF-α signaling. In present study, we took advantage of primary human nucleus cells and mimic *in vivo* stimulation. We found Atsttrin significantly down-regulated TNF-α-induced inflammatory cytokines’ expression at transcriptional level. Sequentially, the protein expressions of inflammatory molecules were dramatically reduced. Collectively, Atsttrin has a therapeutic effect in IDD, and this effect is through inhibiting TNF-α signaling.

Atsttrin is a new engineered molecule derived from the growth factor PGRN. Nowadays, more and more studies indicated Atsttrin exhibited a strong anti-inflammation ability and exerted a protective effect in some pathological processes. It’s reported Atsttrin was therapeutic in inflammatory arthritis mice model; moreover, overexpression of Atsttrin in local articular cartilage protected against osteoarthritis [13, 36]; and study showed that Atsttrin promoted bone healing through inhibiting TNF-α pathway in the physiological process. All these previous studies demonstrated that Atsttrin could be a drug candidate for the inflammatory disease in which TNF-α plays a predominate role. And indeed, in our present study, we found Atsttrin exerted a protective effect through, at least partially through inhibiting TNF-α signaling.

Collectively, Atsttrin is therapeutic in the intervertebral disc degenerative disease via targeting TNF-α and Atsttrin could be a noval drug candidate for the inflammatory degenerative diseases.

## MATERIALS AND METHODS

### Ethics statement

40 patients with single level lumbar disc herniation were enrolled between 2017.1.1 and 2017.5.31 at Qilu Hospital of Shandong University. Collection of lumbar disc tissue and peripheral blood samples was performed according to the medical ethics regulations of Medical Ethical Committee of Qilu Hospital of Shandong University. This research was approved by the Qilu Hospital of Shandong University Review Board, and written informed consent document was requested and received from all patients and healthy individuals in the study. The animal experimental procedures were performed with the formal approval of The International Guiding Principles for Animal Research, as approved by the Laboratory Animal Centre of Shandong University.

### Patients

All the patients enrolled in this study signed the informed consent and agreed to participate in this study, 40 patients with lumber disc herniation (LDH) (37–61 years; mean 51.63 ± 9.12 years) were enrolled in our study as the experimental group. The selection criteria were as follows: 1) persistent lumbosacral pain accompanied with lower limb radiating pain at least 3 months ,accompanied by or without limb numbness; 2) lumber disc MRI and CT imaging studies showed significant disc herniation performance and compression of the nerve; 3) Lasegue’s sign (+) or positive for femoral nerve traction test (+). The exclusion criteria were as follows: 1) LDH combined with osteoarthritis, rheumatoid osteoarthritis; (2) LDH combined with malignant tumor; (3) recent history of trauma and surgery; and (4) LDH combined with infections of other organs and systems. And we selected 10 normal people (24-25 years; mean 24.13 ± 0.85 years) as the control group.

Four milliliters of blood were extracted with a heparin sodium anticoagulant tube from all the people early in the morning after fasting. Blood samples were sent to the laboratory immediately and centrifuged at 4°C at 2,000 g for 10 minutes. Supernatant was extracted and used for the detection of TNF-α.

### Primary cell isolation and culture

All procedures are in compliance with the requirement of Qilu Hospital’s Research Ethic Committee, Shandong University, China. All the intervertebral disc tissues were harvest from the lumber spine surgery of the patients with IDD. After washed with sterile phosphate buffer (PBS) 3 times and then removed the endplate cartilage and annulus fibrosus (AF) carefully using a dissecting microscope. The nucleus pulposus (NP) was digested with 0.25% trypsin (Hyclone, Logan, USA) for 30 minutes and 0.2% type II collagenenzyme (Sigma-Aldrich, St. Louis, USA) for 4 hours. Then seeded the NP cell suspension in the 25 cm^2^ cell culture flask at a density of approximately 25,000cells/cm^2^ in DMEM/F12 culture medium (HyClone, Logan, USA) supplemented with 10% fetal bovine serum (FBS, Gibco, Waltham, USA), 100 U/mL penicillin and 100 mg/mL streptomycin (HyClone, USA) and then incubated in the incubator with 5% CO_2_, 37°C. Replaced the culture medium every 3 days, after the NP cells reached 80-90% confluence then passaged with 0.25% trypsin. In this study, we used second or third generation cells for all experiments.

### Culture of IVD tissues explants

The procedure was performed as previously described [42–44]. The human intervertebral disc (IVD) tissues derived from surgical resection of patients with lumbar fracture. After carefully separated the annulus fibrous and endplates under aseptic conditions then cultured in DMEM/F 12 culture medium (HyClone, Logan, USA) supplemented with 10% fetal bovine serum (FBS, Gibco, Waltham, USA), 100 U/mL penicillin and 100 mg/mL streptomycin (HyClone, USA) in the absence of presence of TNF-α (50 ng/ml, Abcam, USA) with or without Atsttrin (1 μg/ml, Sangon Biotech, China) for 7d, the medium is replaced every 2 days.

The specimen of mouse spine were taken from the 3-month-old mice, after complete dissected the spine and removed the surrounding soft tissues, cut the lumbar spine into the length of 5 mm and keep the integrity of the intervertebral disc. Soaked the lumbar spine of the mouse with 100 U/ml penicillin/streptomycin 30 minutes, then culture the lumbar spine in the medium as described above. For Safranin O/Fast Green staining and HE staining, the explants of lumbar spine of the mouse were fixed in 4% paraformaldehyde for 48 h and processed for paraffin embedding. Sections were stained with Modified Safranine O-Fast Green FCF Cartilage Stain Kit (Solarbio, USA) according to the manufacture instruction.

### Immunohistochemistry (IHC) and immunofluorescence staining

Degenerated IVD tissues obtained from the patients with lumber disc herniation, the disc samples were fixed in 4% formaldehyde and then embedded with paraffin. The paraffin was cut into 4 um sections. After deparaffinized and rehydrated, 0.125% trypsin (ZSGB-Bio, Beijing, China) was used to antigen repair, then incubated the section with 3% hydrogen peroxide to eliminate endogenous peroxidase activity. Then use 20% goat serum (ZSGB-Bio, Beijing, China) to close the nonspecific protein binding sites. The sections were incubated with rabbit anti-human iNOS (1:200, Abcam, USA), rabbit anti-human MMP-13(1:200, Abcam, USA) and rabbit anti-human COX-2 (1:200, Abcam, USA, rabbit anti-human IL-17(1:200, Affinity, USA)) primary antibodies overnight at 4°C. Then the incubated the sections with a goat anti-rabbit immunoglobulin(IgG)-horseradish peroxidase (HRP) secondary antibody (1:200, ZSGB-Bio, China) at 37°C for 1 hour. IX71-SIF type microscope (Olympus, Tokyo, Japan) was used to take the images, image-Pro Plus software (Media Cybernetics, Warrendale, USA) was used to quantify the mean densities (MDs) of the expression intensities of all the cytokines.

For immunofluorescence staining The NP cells were treated with 10 ng/ml of TNF-α (Abcam, USA) in the presence or absence of 200 ng/ml of Atsttrin (Sangon Biotech, China) for 48 h. Then fixed the cells with 4% formaldehyde for 10 minutes, and permeabilized in 0.2% Triton-X 100 for 15 minutes and then blocked in 1% BSA for 30 minutes at room temperature. Then incubate the cells with rabbit anti-human COX-2 (1:400, Abcam, USA) and rabbit anti-human iNOS (1:400, Abcam, USA) primary antibody overnight at 4°C. The next day cells were washed with PBS and then incubated in a secondary fluorescently labeled goat anti-rabbit immunoglobulin (IgG) antibody (1:200, ZSGB-BIO, China) for 1 hour at room temperature. The image was taken by a fluorescent microscope (Olympus IX51, Japan).

### Cell treatment

To investigate the effects of Atsttrin on TNF-α, human NP cells were seeded into a six-well plate and stimulated with TNF-α (10 ng/ml) with or without Atsttrin (0, 20, 50, 100, 200 ng/ml, Sangon Biotech, China) for 48 hours. Then harvest the NP cells for western blot analysis and real-time PCR (RT-PCR) to observe the expression level of COX-2, MMP-13 and iNOS. The supernatant were harvested for ELISA test of IL-6.

### Protein extraction and western blotting

After the NP cells were confluence in the six-well plate then lysed the cells with RIPA buffer (Millipore, Billerica, MA, USA) on ice for 40 minutes. Then centrifuged at 4°C, 12,000 rpm. Carefully absorb the supernatant and measure the protein concentration with BCA protein Assay Kit (Biotechnology Co., Beijing, China) according to the manufacture instructions. Separated each group of protein in 10% acrylamide–SDS–PAGE and subsequently transferred to PVDF membranes (Millipore, USA). Blocked the membranes with TBST which contain 5% milk powder and followed incubate with primary antibodies (rabbit anti-MMP-13, 1: 1,000, Abcam, USA; rabbit anti-COX-2, 1:1,000, Abcam, USA and rabbit anti-iNOS, 1:1000, Ancam) overnight at 4°C. Then incubate the PVDF membranes in goat anti-rabbit immunoglobulin (IgG)-horseradish peroxidase (HRP) secondary antibody (1:3000; Beijing Golden Bridge Bioechnology, China) at room temperature for 1 hour. Equal protein loading was confirmed by reprobing the membranes with the rabbit anti-GAPDH-HRP antibody (1:3,000, Proteintech, USA). Protein bands were detected using a FluorChem E Chemiluminescent Western Blot Imaging System (Amersham Imager 600, GE Amersham USA) and image-Pro Plus software (Media Cybernetics, Warrendale, USA) was used to analyze the optical density of each strip [23].

### RNA extraction and real-time PCR

After treated with TNF-α and Atsttrin, the human NP cells were disintegrated with TRIzol reagent (Takara Bio, Japan). After total RNA was extracted then reverse transcribed to cDNA according to the manufacturer’s instructions of cDNA Synthesis Kit (GeneCopoeia, Inc. USA). Real-time PCR reactions were carried out on Roche LightCycler (Roche, USA) utilizing a SYBR Green PCR Matrix Mix (Toyobo, Japan). The nucleotide sequences of MMP-13, COX-2, iNOS and GAPDH primers are listed in Table 1.

**Table 1:** Real-time PCR primers

Target	Forward primers, 5′–3′	Reverse primers, 5′–3′
**COX-2**	TCAGCCATACAGCAAATCCTTG	GTCCGGGTACAATCGCGACTT
**MMP-13**	CATGAGTTCGGGCCACTCCTT	CCTGGACCATAGAGAGACTGGA
**iNOS**	CGTGGAGACGGGAAAGAAGT	FACCCCAGGCAAGATTTGGA
**GAPDH**	GCACCGTCAAGGCTGAGAAC	TGGTGAAGACGCCAGTGGA

Abbreviations: MMP13: matrix metalloprotease 13; COX-2: cyclooxygenase-2; iNOS: inducible nitric oxide synthase.

### Enzyme-linked immunosorbent assay (ELISA)

We obtained the serum sample from the patients with LDH and stored at −20°C. The supernatants harvested from NP cell culture and stored at −20°C until assay. The serum concentration of TNF-α was measured by ELISA according to the manufacturer’s instructions (Abcam, USA), IL-6 in the supernatants from conditioned NP cells cultures were detected utilizing Human IL-6 Elisa kit (BlueGene, China) follow the instructions protocol. The color reaction was assayed at 450 nm using a Varioskan flash multifunction plate reader (Thermo Fisher Scientific, Waltham, MA).

### Statistical analysis

Statistics were analyzed using SPSS 22.0 (SPSS, Chicago, Illinois, USA), all data were conducted as mean values ± standard error of mean (SEM). P<0.05 was considered as statistically significant.

## Abbreviations

PGRN: progranulin
HE staining: Hematoxylin and Eosin staining
TNF-α: Tumor Necrosis Factor α
IL-1β: interleukin-1β
IVD: intervertebral disc
MMP13: matrix metalopeptidase 13
COX-2: cyclooxygenase-2
iNOS: inducible nitric oxide synthase
NP: nucleus pulposus
AF: annulus fibrosus
